# Supplementary material for: Morphological and Molecular Identification of Sarcocystis arctica in Captive Cheetahs (Acinonyx jubatus) in China Helps Clarify Phylogenetic Relationships with Sarcocystis caninum and Sarcocystis felis
Source: Animals (Basel). 2025 Jan 10;15(2):180. doi: 10.3390/ani15020180 (PMC11758320; doi:10.3390/ani15020180)
Supplement: Supplementary file 1 [file animals-15-00180-s001.zip › animals-3347724-supplementary.pdf]

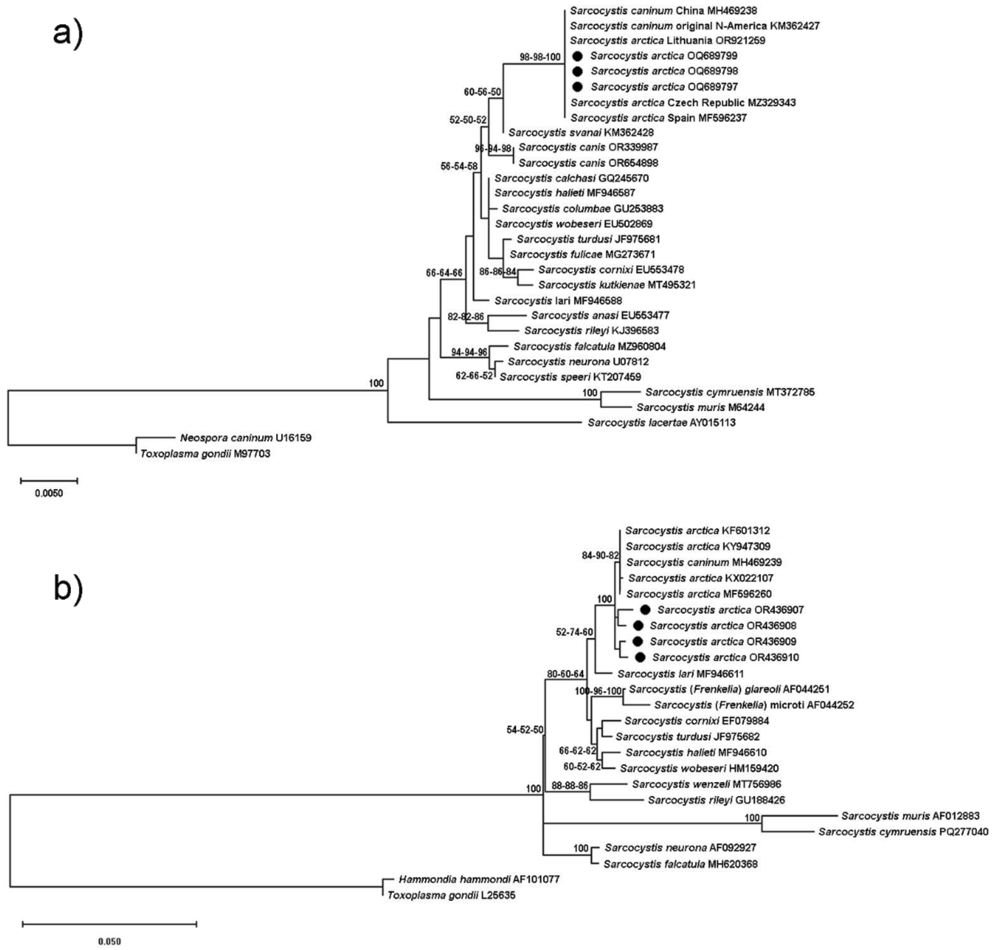

Supplement Figure S1. Maximum likelihood (ML) phylogenetic trees inferred from nuclear 18S rRNA (a) and 28S rRNA (b) sequences. The GenBank accession numbers of all the sequences included in the analyses are given behind the taxon names. The values attached to branches represent percent bootstrap values per 1000 replicates (only values ≥50% are shown), whereby the results of three independent analyses (i.e., alignments) are indicated. Species of Toxoplasmatinae served as outgroups. The new nucleotide sequences of *Sarcocystis arctica* from cheetahs are highlighted by black symbols.
